# Correction: Dynamic linkages between renewable energy, carbon emissions and economic growth through nonlinear ARDL approach: Evidence from Iran

**DOI:** 10.1371/journal.pone.0258612

**Published:** 2021-10-11

**Authors:** M. S. Karimi, S. Ahmad, H. Karamelikli, D. T. Dinç, Y. A. Khan, M. T. Sabzehei, S. Z. Abbas

There are errors in the Funding statement. The correct Funding statement is as follows: The authors received no specific funding for this work.
